# PM-Bound Polycyclic Aromatic Hydrocarbons and Nitro-Polycyclic Aromatic Hydrocarbons in the Ambient Air of Vladivostok: Seasonal Variation, Sources, Health Risk Assessment and Long-Term Variability

**DOI:** 10.3390/ijerph19052878

**Published:** 2022-03-01

**Authors:** Yan Wang, Hao Zhang, Xuan Zhang, Pengchu Bai, Andrey Neroda, Vassily F. Mishukov, Lulu Zhang, Kazuichi Hayakawa, Seiya Nagao, Ning Tang

**Affiliations:** 1 Graduate School of Medical Sciences, Kanazawa University, Kanazawa 9201192, Japan; wangyan@stu.kanazawa-u.ac.jp (Y.W.); zhanghao@stu.kanazawa-u.ac.jp (H.Z.); zhangxuan@stu.kanazawa-u.ac.jp (X.Z.); baipengchu@stu.kanazawa-u.ac.jp (P.B.); 2 Pacific Oceanological Institute, Far Eastern Branch, Russian Academy of Sciences, 690041 Vladivostok, Russia; aneroda@poi.dvo.ru (A.N.); vmishukov@poi.dvo.ru (V.F.M.); 3 Institute of Nature and Environmental Technology, Kanazawa University, Kanazawa 9201192, Japan; zhang-lulu@se.kanazawa-u.ac.jp (L.Z.); hayakawa@p.kanazawa-u.ac.jp (K.H.); seiya-nagao@se.kanazawa-u.ac.jp (S.N.); 4 Institute of Medical, Pharmaceutical and Health Sciences, Kanazawa University, Kanazawa 9201192, Japan

**Keywords:** persistent organic pollutants, atmospheric transport, traffic emission, Vladivostok

## Abstract

Total suspended particles (TSP) were collected in Vladivostok, Russia, which is a typical port city. This study investigated the concentration, potential sources, and long-term variation in particle PAHs and NPAHs in the atmosphere of Vladivostok. The PAH and NPAH concentrations were higher in winter than in summer (PAHs: winter: 18.6 ± 9.80 ng/m^3^ summer: 0.54 ± 0.21 ng/m^3^; NPAHs: winter: 143 ± 81.5 pg/m^3^ summer: 143 ± 81.5 pg/m^3^). The diagnostic ratios showed that PAHs and NPAHs mainly came from vehicle emissions in both seasons, while heating systems were the main source of air pollution in winter. The TEQ assessment values were 2.90 ng/m^3^ and 0.06 ng/m^3^ in winter and summer, respectively, suggesting a significant excess cancer risk in the general population in winter. The ILCR values conveyed a potential carcinogenic risk because the value was between 1 × 10^−5^ and 1 × 10^−7^ and ingestion was a main contributor in Vladivostok. However, it is worth noting that the concentrations of PAHs and NPAHs showed an overall downward trend from 1999 to 2020. An important reason for this is the cogenerations project implemented by the Far Eastern Center for Strategic Research on Fuel and Energy Complex Development in 2010. This research clarified the latest variations in PAHs and NPAHs to provide continuous observation data for future chemical reaction or model prediction research.

## 1. Introduction

Atmospheric particulate matter (PM) is a ubiquitous air pollutant that affects flora, fauna and humans. Although the concentrations of polycyclic aromatic hydrocarbons (PAHs) and nitrated polycyclic aromatic hydrocarbons (NPAH) in PM are generally much lower than those of other compounds [1,2], PAHs and NPAHs have been found to be the major components associated with carcinogens and mutagens, such as BaP and 6-nitrochrysene (6-NC) [3,4]. PAHs and NPAHs can easily move among ecosystems and adversely affect many organisms, including humans, due to their small size and persistence [5]. PAHs and NPAHs mainly originate from anthropogenic pyrogenic sources, such as coal combustion, vehicle emissions and biomass burning [6,7,8]. In fact, PAHs and NPAHs are ubiquitous in the air environment worldwide, even in remote regions, because of long-range atmospheric transportation [9,10,11,12]. In addition, PAHs in the atmosphere undergo a series of complex chemistry reactions that generate more PAH derivatives, for example, NPAHs not only come from incomplete combustion processes, but also form during the transformation process facilitated by photochemical reactions, hydroxyl radicals, nitrate radicals and ozone [13]. Nevertheless, 2-nitropyrene (2-NP) and 2-nitrofluoranthene (2-NFR) are produced only by heterogeneous reactions caused by free radicals [14]. Since NPAHs have higher mutagenicity and genotoxicity than PAHs [15,16], species-specific information on the sources of PAHs and NPAHs is essential to assess the health risks of PAHs and NPAHs.

Vladivostok, Russia, is located in the northeast of the East Asian continent and is affected by the East Asian monsoon. According to our previous studies, the East Asian monsoon (EAM) is of great importance to atmospheric pollution in East Asia and the surrounding regions [17,18,19]. The warm–wet East Asian summer monsoon (EASM) and the cold–dry East Asian winter monsoon (EAWM) are two major components of the EAM. During winter, the EAWM can force Asian continental air masses to cross Mongolia, Russia, China, the Korean Peninsula and Japan to the North Pacific Ocean, which moves large numbers of contaminated air masses and dust particles [20]. In contrast, the concentration of atmospheric pollution is mainly influenced by local pollution and clean ocean air masses during the EASM [21,22]. In addition, the Free Port of Vladivostok has had a significant increase in cargo turnover, causing traffic emissions to play an important and exceptional role in air pollution over the last 20 years and to participate in the circulation system of air pollution in East Asia [23,24].

It is worth noting that the energy structure at Vladivostok has dramatically changed in recent years. The residential heating period lasts appromixmately six months every year. Our previous study in Vladivostok demonstrated that coal combustion was the major source of ambient PAHs and NPAHs in winter [25,26]. This study also showed that human activities and local government energy policies directly influence the concentrations and compositions of PAHs and NPAHs. Fortunately, the local government replaced coal heating energy with that of natural gas in 2010 [27]. To better understand the sources, transportation, and potential human cancer risk in Vladivostok, the PAHs and NPAHs in the PM in Vladivostok from 1999 to 2020 were investigated in this study. The objective of this work was to evaluate the pollution status (concentration, composition, main sources) and health risks of PAHs and NPAHs in the atmosphere of Vladivostok through the latest survey results from 2019 to 2020. Additionally, the long-term variability and major influencing factors of the PAHs and NPAHs in the atmosphere of Vladivostok were investigated by comparison with the previous investigation results. This study will help analyse the environmental impact of PAHs and NPAHs in the atmospheres of countries and regions located in the EAM region.

## 2. Materials and Methods

### 2.1. Sample Collection

**Figure 1:**
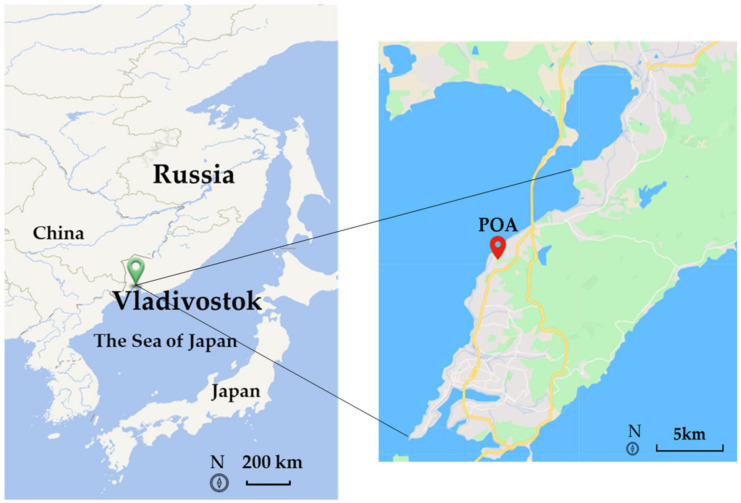
Sampling location of Vladivostok (43.20° N, 131.93° E). Vladivostok is shown by green mark on left map and POA located in Vladivostok is shown by red mark on right map.

Thirty-three total atmospheric samples were collected from December 2019 to July 2020 in the Pacific Oceanological Institute (POI), Far Eastern Branch of Russian Academy of Sciences, Vladivostok, Russia (43.20° N, 131.93° E), as shown in Figure 1. According to our previous studies, Vladivostok can be influenced by domestic pollutants in summer, while can be influenced by long-range transportant pollutants in winter [28]. A high-volume air sampler (Sibata Sci. Tech. Ltd., Saitama, Japan) equipped with a quartz membrane filter (2500QAT-UP, Pallflex Products, Show Low, AZ, USA) was used to collect TSP samples at an intake flow rate of 1000 L/min. Details on the sampling periods and sample numbers are listed in Table S1. After sampling, the samples were placed in a drying cabinet for 48 h, and then refrigerated at −20 °C until the samples were analyzed.

### 2.2. PAHs and NPAHs Analysis

Ten PAHs, including fluoranthene (FR), pyrene (Pyr), benzo[*a*]anthracene (BaA), chrysene (Chr), benzo[*b*]fluoranthene (BbF), benzo[*k*]fluoranthene (BkF), benzo[*a*]pyrene (BaP), benzo[*e*]pyrene (BeP), benzo[*ghi*]perylene (BgPe), and indeno [1,2,3-*cd*]pyrene (IDP) (Supelco Park, Bellefonte, PA, USA) and six NPAHs, 2-NFR, 1-,2-NPs, 6-nitrochrysene (6-NC), 7-nitrobenz[*a*]anthracene (7-NBaA), and 6-nitrobenzo[*a*]pyrene (6-NBaP) (Chiron As, Trondheim, Norway), in each PM sample were detected using our method [29]. The U.S. EPA 610 PAH mix, including the ten target PAHs, was purchased from Dr. Ehrenstorfer GmbH (Augsburg, Germany). Two internal standards (Pyr-d10 and BaP-d12) were purchased from Wako Pure Chemicals (Osaka, Japan) and those for NPAHs (2-NFR-7-NBaA) were purcased from Chiron AS. In brief, each filter was cut into small pieces and placed in a flask. After adding Pyr-d10 and BaP-d12 (Wako Pure Chemicals, Osaka, Japan), compounds successively underwent ultrasonic extraction, washing, concentration, dilution and filtration. Then, PAHs and NPAHs in the solution were analyzed by a high-performance liquid chromatographic system with fluorescence detection (Shimadzu Inc., Kyoto, Japan). All others reagents were analytical reagent grade. The methods employed for pretreatment, quantitative determination and quality control are detailed in our previous study [30]. In addition, weather conditions, including temperature, humidity and mean wind speed during each period at Vladivostok were obtained from the National Centers for Environmental Information of National Oceanic and Atmospheric Administration (NOAA) (https://www.ncei.noaa.gov/ (assessed 20 May 2021)).

### 2.3. Health Risk Assessment

The toxicity equivalent factor (TEF) model is usually used for calculations of PAH carcinogenic risk based on the procedure indicated by the United States Environmental Protection Agency (USEPA) (2016) [31,32]. The development and establishment of the TEF model for PAHs could aid in the precise characterization of the carcinogenic properties of PAH mixtures. Generally, benzo[*a*]pyrene (BaP) is believed to be the most toxic PAH and has been well-characterized toxicologically [33,34]. Numerous studies have shown that it is necessary to systematically measure BaP as an indicator of the PAH group, causing it to be a major health risk driver [35,36]. Therefore, BaP was designated as the reference compound in the calculation of the toxicity of PAHs using the TEF model. The toxicity equivalency (*TEQ*) calculations were performed as follows [37,38]:

(1) $$TEQ_{i}=C_{i}\times TEF_{i}\;$$

(2) $$TEQ_{total}=\sum TEQ_{i}\;$$

where *TEF_i_* is the toxic equivalent factor of individual PAHs (Table S2).

The incremental lifetime cancer risk (ILCR) of individual PAHs and NPAHs is mainly based on animal laboratory tests and occupational epidemiological studies [39]. The ILCR estimation of PAHs and NPAHs has many uncertainties due to many factors, such as toxic synergy, in which most PAHs occur together with other carcinogenic pollutants. In addition, individuals exposed to different environments from different exposure pathways should be considered. In this study, the ILCR was assessed by applying the USEPA standard model.

The ILCRs of ingestion, dermal absorption and inhalation were calculated as follows [40,41]:

(3) $$R_{ing}=\frac{C\times CSF_{ing\;}\times \sqrt[{3}]{\frac{Bw}{70}}\times IR_{ing}\times EF\times ED}{BW\times AT\times 10^{6}}$$

(4) $$R_{inh}=\frac{C\times CSF_{inh}\times \sqrt[{3}]{\frac{Bw}{70}}\times IR_{inh}\times EF\times ED}{BW\times AT\times PEF}$$

(5) $$R_{dem}=\frac{C\times CSF_{dem}\times \sqrt[{3}]{\frac{Bw}{70}}\times \;\mathrm{SA}\;\times \;\mathrm{AF}\;\times \;\mathrm{ABS}\;\times EF\times ED}{BW\times AT\times 10^{6}}$$

where the risk values for ingestion, inhalation and dermal exposures are expressed by *R_ing_*, *R_inh_*, and *R_dem_*, respectively; *C* is equal to the sum of the toxic equivalent concentrations of the 16 individual PAHs in ng/m^3^ (*C* = *TEQ_PAH_*); *CSF_ing_*, *CSF_inh_* and *CSF_dem_* are the carcinogenic slope factors for ingestion, inhalation and dermal exposures, respectively, in mg/kg/day; *BW* is the average body weight in kg; *IR_ing_* is the intake rate under the ingestion exposure route in mg/day; *EF* is the annual exposure frequency in day/year; *ED* is the duration of exposure in years; *AT* is the average lifespan in days; *IR_inh_* is the rate of intake under inhalation exposure in mg/day; *PEF* is the particle emission factor in mg/kg; *SA* is the exposed area of the skin in cm^2^; *AF* is the skin adherence factor in mg/cm^2^; and *ABS* is the skin absorption factor in day^−1^. The carcinogenic slope factors (CSFs) based on the cancer-causing ability of BaP were parameterized as 7.3, 25, and 3.85 (1/(mg/kg/day)) for ingestion, inhalation and dermal exposure, respectively [42]. For the related values, refer to Table S3 [43,44].

The total *ILCR* is the summation of the three different forms of risk [45].

(6) $$Total\;ILCR=ILCR_{ing}+ILCR_{inh}+ILCR_{dem}\;$$

### 2.4. Statistical Analysis

SPSS version 24.0 (IBM Corp., Armonk, NK, USA) was used for the statistical analysis of the data. T-tests were used to compare the relationships between PAHs and NPAHs in the PM samples and seasons. The correlations between PAHs, NPAHs and meteorological conditions were assessed using the Spearman correlation coefficient (two-tailed). A *p* value of less than 0.05 indicated a statistically significant result.

## 3. Results

### 3.1. Concentrations of PAHs and NPAHs

Table 1 presents the average concentration ± standard deviation of ten PAHs (ΣPAHs) and six NPAHs (ΣNPAHs) in winter and summer. The average concentrations of ΣPAHs and ΣNPAHs in winter and summer were 18.6 ± 9.80 ng/m^3^ and 0.54 ± 0.21 ng/m^3^, respectively. The average concentrations of ΣNPAHs in winter and summer were 143 ± 81.5 pg/m^3^ and 8.92 ± 3.97 pg/m^3^, respectively. Consistent with our previous findings [26], the airborne PAH and NPAHs in winter were higher than those in summer (ρ < 0.01). As the most abundant PAH, BgPe contributed 21.9% and 31.5% of the total PAHs in winter and summer, respectively, in accordance with our last survey [28]. It is worth noting that the BaP concentration was 2.14 ± 1.35 ng/m^3^ in winter, which still exceeded the target value (1 ng/m^3^) in Europe, consistent with what has been found previously. For NPAHs, 2-NFR (30%) exhibited the highest proportion in winter, and 7-NBaA (48%) (representing the first time this component had been analyzed in Vladivostok) exhibited the highest proportion in summer, because 7-NBaA can be emitted from gasoline vehicles as well as from atmospheric reactions [46]. The reason for the high 7-NBaA concentration in summer needs to be further analyzed. In both seasons, 2-NP (winter: 2%, summer: 3%) was the least abundant NPAH.

In addition, local meteorological conditions could significantly impact the PAHs and NPAHs in the atmosphere. By the Spearman correlation coefficient, the concentrations of PAHs and NPAHs were negatively correlated with temperature (*p* < 0.01, as shown in Tables S4 and S5). In addition to PAHs and NPAHs with higher vapour pressures conducting gas/particle partitioning via a change in temperature [47], the changes in the main sources in winter, summer and reactivity were the main factors causing this correlation [48] (see Section 3.2 for details). However, no significant correlation with wind speed or humidity was found during the entire sampling period. This might be due to by the seasonal difference in wind speed and humidity not being significant (Tables S4 and S5).

### 3.2. Main Sources of PAHs and NPAHs

**Figure 2:**
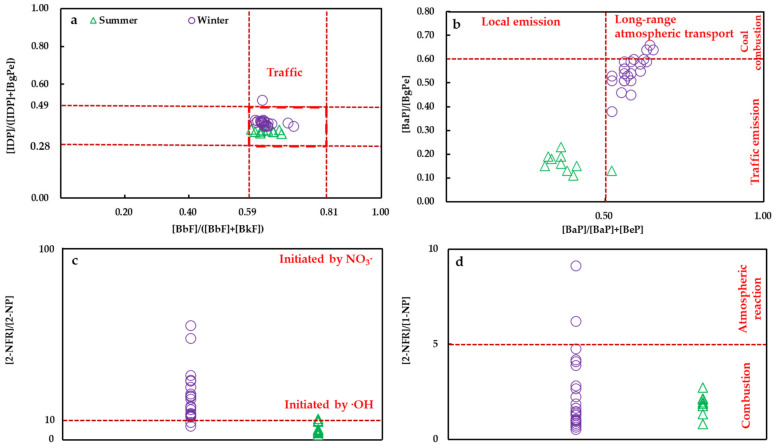
The diagnostic ratios of PAHs and NPAHs at Vladivostok in both seasons and their specific sources. (**a**) diagnostic [BbF]/([BbF] + [BkF]) and [IDP]/([BgPe] + [IDP]) ratios from traffic emission; (**b**) diagnostic [BaP]/([BaP] + [BeP]) and [BaP]/[BgPe] ratios from local emission, long-range transportation, traffic emission, coal combustion; (**c**) diagnostic [2-NFR]/[2-NP] ratios from gas-phase chemical reaction, (**d**) diagnostic [2-NFR]/[1-NP] ratios from combustion or atmospheric reaction.

The chemical compositions of pollution provide a better understanding of pollution sources and their environmental impacts [49,50]. Many studies have used different PAH and NPAH diagnostic ratios as tracers to distinguish their diverse sources [51,52]. The latest research shows that [BbF]/([BbF] + [BkF]) and [IDP]/([BgPe] + [IDP]) have higher accuracy, are not affected by spatial and temporal distributions, and can be used to distinguish PAHs mainly from traffic sources or other sources [53]. As shown in Figure 2a, almost all values of [BbF]/([BbF] + [BkF]) and [IDP]/([BgPe] + [IDP]) were in the ranges of 0.67–0.81 and 0.26–0.49, respectively. These results suggest that vehicle emissions were the main source of PAHs and NPAHs in Vladivostok from 2019 to 2020. On the other hand, [BaP]/[BgPe] can be used to distinguish PAHs and NPAHs from traffic sources (<0.6) or coal combustion s (>0.6) [54]. The [BaP]/([BaP] + [BeP]) ratio has been used to assess the contributions of local sources to atmospheric PAHs and NPAHs (>0.6) [55]. As shown in Figure 2b, most of the [BaP]/[BgPe] ratios were less than 0.6, which also showed that traffic sources are the main contributors to airborne PAHs and NPAHs in Vladivostok. However, some of the [BaP]/[BgPe] ratios in winter were close to or higher than 0.6, indicating that coal combustion is still a nonnegligible contributor in winter. The values of [BaP]/([BaP] + [BeP]) mainly varied between 0.52 and 0.65 in winter, indicating a dominant contribution from long-range atmospheric transport. According to other studies, the contaminated air mass may come from Northeast China and Mongolia [28,56]. In summer, almost all [BaP]/([BaP] + [BeP]) ratios were below 0.5, indicating that local emissions might contribute to PAHs and NPAHs at Vladivostok. As in our previous study [1,11,28], this may be a reason for the decrease in [BaP]/([BaP] + [BeP]), since the air mass in summer mainly comes from the Vladivostok port.

Additionally, NPAHs in ambient air are either from primary emissions or from secondary formations. Laboratory studies and field observations have shown that 2-NP and 2-NFR are formed during the photochemical transformation of emitted PYR and FR by atmospheric oxidants [57,58,59], while 1-NP is emitted from vehicle exhausts [60,61]. When the concentration ratio of [2-NFR]/[2-NP] is approximately 10, 2-NP and 2-NFR are from the photochemical by the hydroxyl radical pathways, whereas when the [2-NFR]/[2-NP] concentration ratios are approximately 100, 2-NP and 2-NFR are from the reaction for gas-phase nitrate radical reactions [62,63]. Figure 2c reveals that most of the concentration ratios of [2-NFR]/[2-NP] approached 10 in both seasons, indicating that 2-NP and 2-NFR were mainly formed by hydroxyl radical-initiated photochemical reactions. [2-NFR]/[1-NP] can be divided into airborne NPAHs that are mainly generated from combustion (fresh particles, value < 5) or atmospheric reactions (ageing particles, value > 5) [64]. As shown in Figure 2d, almost all ratios were less than or close to 5, indicating that the NPAHs except 2-NP and 2-NFR were mainly from the incomplete combustion of fossil fuels and biomass in both seasons.

### 3.3. Health Effects of PAHs and NPAHs

#### 3.3.1. Toxic Equivalency Relative to BaP (TEQ)

Table 2 presents the results obtained from the TEQ for total and individual PAHs and NPAHs (except 2-NP, 7-NBaA, 6-NBaP due to a lack of toxic equivalent factors data) in winter and summer. In winter, the average TEQ of the total PAHs and NPAHs(ΣPAHs + ΣNPAHs) was 2.90 ng/m^3^. During summer, the average TEQ of (ΣPAHs + ΣNPAHs) was 0.06 ng/m^3^. The average TEQ concentration of (ΣPAHs + ΣNPAHs) in winter was approximately three times higher than the European Union standard (1 ng/m^3^) [65], indicating that the atmosphere in Vladivostok is still seriously contaminated in winter. These results also showed that BkF, BbF, BaP and IDP were the dominant components of the total average TEQ concentrations in both seasons (winter: 93%, summer: 76%). The International Agency for Research on Cancer (IARC) has classified BaP as a probable carcinogen and BkF, BbF, and IDP as possible carcinogens to humans. The concentration of TEQ in NPAHs was mainly determined by 6-NC because the carcinogenic effect of 6-NC is much higher than that of BaP [66], and the proportions of [6-NC]/[NPAHs] in winter and summer were 95% and 99%, respectively.

#### 3.3.2. ILCR Assessment

To further understand the health risks for the different sexes, we analyzed the ILCR and total accumulated risks. The ILCR collectively accounts for the integrated lifetime risks of exposure to airborne PAHs and NPAHs through direct ingestion, dermal contact, and inhalation. The USEPA recommends an acceptable carcinogenic risk value of 1.0 × 10^−6^, while there is a higher probability of cancer occurring from PAH and NPAH exposure if the value exceeds 1.0 × 10^−4^ [67,68,69,70]. As shown in Table 3, the total ILCRs of PAHs and NPAHs from the three exposure routes were exhibited the following order: ingestion exposure (male: winter: 1.36 × 10^−5^, summer: 2.96 × 10^−7^; female: winter: 1.23 × 10^−5^, summer: 2.68 × 10^−7^) > dermal exposure (male: winter: 3.71 × 10^−6^, summer: 8.10 × 10^−8^; female: winter: 2.68 × 10^−7^, summer: 7.33 × 10^−8^) > inhalation exposure (male: winter: 6.83 × 10^−9^, summer: 1.49 × 10^−10^; female: winter: 6.19 × 10^−9^, summer: 1.35 × 10^−10^). In winter, of the three exposure routes, ingestion exposure and dermal exposure exceeded the acceptable carcinogenic risk value. Compared with other studies [71,72], these results showed that people living in Vladivistok were exposed to PAHs via ingestin and dermal contact at a level posing a potential risk to their health.

Numerically, the calculated male’s ILCRs were higher than the female’s ILCRs in both seasons, as shown in Table 3. In fact, the ILCRs for males remained on the same order of magnitude as the female’s ILCRs. The slight difference may be due to the lower body weight and the greater sensitivity to toxic substances of females [40].

The ILCRs of PAHs and NPAHs from all exposure routes were much higher in winter than in summer. The ranges of the ILCR of PAHs and NPAHs from all exposure routes were 6.19 × 10^−9^–1.36 × 10^−5^ and 1.35 × 10^−10^–2.96 × 10^−7^ in winter and summer, respectively. In winter, the values of the ILCRs of PAHs and NPAHs exceeded the acceptable carcinogenic risk value of USEPA, indicating that long-term residents in Vladivostok are exposed to potential carcinogenic risks. Likewise, the treatment of pollution sources in winter is still an urgent task.

### 3.4. Long-Term Trends and Variations in Σ9PAHs and 1-NP

**Figure 3:**
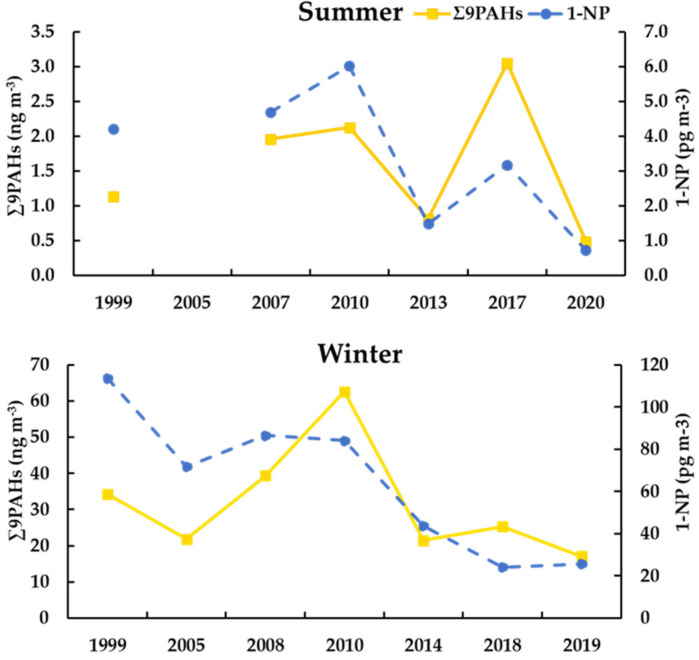
Yearly variations in ∑9PAHs (except BeP) and 1-NP at Vladivostok in summer and winter.

Figure 3 presents the temporal variations in Σ9PAHs (except BeP) and 1-NP during winter and summer from 1999 to 2020. These compunds have been analyzed since 1999. The Σ9PAHs concentration varied from 17.0 ng/m^3^ (2019) to 34.0 ng/m^3^ (1999) during winter and 0.50 ng/m^3^ (2020) to 3.10 ng/m^3^ (2017) in summer. The 1-NP concentration varied from 24.0 pg/m^3^ to 114 pg/m^3^ in winter and 6.00 pg/m^3^ to 0.70 pg/m^3^ in summer. In a comparison to the concentration of Σ9PAHs in 1999, that of 2019–2020 showed a singnificant decline: 50% in winter and 57% in summer. The concentration of 1-NP decreased by 77% in winter and 83% in summer from that in 1999. Nevertheles, there was a significant increasing trend during both seasons in 2010 and summer in 2017. Based on our previous research, according to the Asia Pacific Economic Cooperation (APEC) meeting in 2012, many infrastructure construction projects were under construction in Russia from 2007 to 2012, which may have led to an increase in the concentration of PAHs [25]. In addition, the Russia–China naval activities in the Sea of Japan and the Sea of Okhotsk in September 2017 [73] may have led to an increase in ∑PAHs, and the impact of these emergencies on PAHs cannot be ignored.

For the control of air pollution, the Russian government has taken many positive steps. Among them, the cogeneration (combined heat and power) project promoted by The Far Eastern Center for Strategic Research on Fuel and Energy Complex Development in 2010 may promote a reduction in PAHs concentrations, and has played an important role in the recent improvement in the atmospheric environment [24,74].

## 4. Conclusions

Vladivostok is an important port city in the Far East of Russia, and participates in the atmospheric cycle of air pollutants in East Asia. We have sequentially conducted investigations and research in Vladivostok for approximately 20 years. In the study, three kinds of target NPAHs were added, and 7-NBaA showed the highest level in summer. Additionally, the risk assessment of different PAH and NPAH exposure routes was carried out first, and it was noted that ingestion and dermal exposure were important exposure routes endangering the health of residents. In this study, the concentrations of PAHs and NPAHs decreased significantly compared with those in 1999. This is because of the cogeneration project implemented by the Far Eastern Center for Strategic Research on Fuel and Energy Complex Development in 2010. However, the concentrations of atmospheric PAHs and NPAHs in winter are still worthy of attention because the average TEQ concentration of ΣPAHs + ΣNPAHs was still approximately three times higher than the value of the European Union standard. The data obtained from this study not only play a role in helping the formulation of atmospheric environmental protection policies in Vladivostok, but also provide basic data for understanding the lon g-range transportation of air pollutants in the East Asian monsoon region.

## Figures and Tables

**Table 1:** Mean concentration ± standard variation of PAHs and NPAHs at Vladivostok in winter and summer from 2019 to 2020.

Compound	Winter	Summer
FR	1.72 ± 0.84	0.02 ± 0.01
Pyr	1.58 ± 0.74	0.02 ± 0.01
BaA	0.92 ± 0.47	0.01 ± 0.01
Chr	1.25 ± 0.57	0.03 ± 0.01
BbF	1.76 ± 0.95	0.05 ± 0.03
BkF	1.01 ± 0.57	0.03 ± 0.01
BaP	2.14 ± 1.35	0.03 ± 0.01
BeP	1.46 ± 0.80	0.05 ± 0.03
BgPe	4.08 ± 2.33	0.20 ± 0.06
IDP	2.68 ± 1.55	0.11 ± 0.04
ΣPAHs (ng/m^3^)	18.6 ± 9.80	0.54 ± 0.21
2-NFR	42.5 ± 31.3	1.29 ± 0.91
1-NP	25.7 ± 16.3	0.73 ± 0.37
2-NP	3.28 ± 3.81	0.28 ± 0.06
6-NC	5.70 ± 3.72	1.12 ± 0.58
7-NBaA	39.5 ± 24.2	4.52 ± 2.46
6-NBaP	26.7 ± 14.7	1.42 ± 0.55
ΣNPAHs (pg/m^3^)	143 ± 81.5	8.92 ± 3.97

**Table 2:** Toxic equivalent concentrations relative to BaP of PAHs and NPAHs (except 2-NP, 7-NBaA, 6-NBaP) at Vladivostok in winter and summer from 2019 to 2020.

		Winter	Summer
PAHs (ng/m^3^)	FR	0.002	0.00002
	Pyr	0.002	0.00002
	BaA	0.092	0.00133
	Chr	0.012	0.00028
	BbF	0.176	0.00482
	BkF	0.101	0.00256
	BaP	2.138	0.02992
	BeP	0.003	0.00011
	BgPe	0.041	0.00198
	IDP	0.268	0.01078
	ΣPAHs	2.84	0.05
NPAHs (pg/m^3^)	2-NFR	0.425	0.01292
	1-NP	2.571	0.07282
	6-NC	57.012	11.24783
	ΣNPAHs	60.01	11.33
Total	ng/m^3^	2.90	0.06

**Table 3:** The ILCR of PAHs and NPAHs according three exposure pathways at Vladivostok in winter and summer from 2019 to 2020.

		Male	Female
Winter (*n* = 23)	Ingestion	1.36 × 10^−5^	1.23 × 10^−5^
	Inhalation	6.83 × 10^−9^	6.19 × 10^−9^
	Dermal	3.71 × 10^−6^	3.36 × 10^−6^
	Total	1.73 × 10^−5^	1.57 × 10^−5^
			
Summer (*n* = 10)	Ingestion	2.96 × 10^−7^	2.68 × 10^−7^
	Inhalation	1.49 × 10-^10^	1.35 × 10^−10^
	Dermal	8.10 × 10^−8^	7.33 × 10^−8^
	Total	3.77 × 10^−7^	3.41 × 10^−7^

## Data Availability

Data is contained within the article or Supplementary Material. The data presented in this study are available in Supplementary Material.

## Supplementary Materials

The following supporting information can be downloaded at: https://www.mdpi.com/article/10.3390/ijerph19052878/s1, Table S1: Sampling periods and sample numbers; Table S2: Toxic equivalent factor (TEF) of polycyclic aromatic hydrocarbons (PAHs) and nitro-PAHs (NPAHs); Table S3: Parameters used for the estimation of the incremental lifetime cancer risks (ILCRs); Table S4. Daily weather conditions in each sample at Vladivostok; Table S5. Spearman correlation coefficient (two-tailed) of each PAHs and NPAHs with weather condition during sampling period.
