# Supplementary material for: PM-Bound Polycyclic Aromatic Hydrocarbons and Nitro-Polycyclic Aromatic Hydrocarbons in the Ambient Air of Vladivostok: Seasonal Variation, Sources, Health Risk Assessment and Long-Term Variability
Source: Int J Environ Res Public Health. 2022 Mar 1;19(5):2878. doi: 10.3390/ijerph19052878 (PMC8910546; doi:10.3390/ijerph19052878)
Supplement: Supplementary file 1 [file ijerph-19-02878-s001.zip › ijerph-1606868-supplementary.pdf]

International Journal of
Environmental Research and Public Health

MDPI

Supplementary Materials:

Table S1. Sampling periods and sample numbers.

Periods (sample numbers)	
**Winter**	**Summer**
2019/12/21-12/30 (9)	2020/06/19-06/22 (3)
2020/01/20-01/27 (7)	2020/07/22-07/29 (7)
2020/02/21-02/28 (7)	

Table S2. Toxic equivalent factor (TEF) of polycyclic aromatic hydrocarbons (PAHs) and nitro-PAHs (NPAHs).

Compound		TEF
PAHs	FR	0.001[b]
	Pyr	0.001[b]
	BaA	0.1[a]
	Chr	0.01[a]
	BbF	0.1[a]
	BkF	0.1[a]
	BaP	1[a]
	BeP	0.002[a]
	BgPe	0.01[b]
	IDP	0.1[a]
NPAHs	2-NFR	0.01[b]
	1-NP	0.1[b]
	6-NC	10[b]

[a] U.S. EPA, 2010. [b] J. F. Collins, J. P. Brown.

Table S3. Parameters used for the estimation of the incremental lifetime cancer risks (ILCRs)

Exposure parameters	Unit	Male	Female
**Body weight (BW)[b]**	Kg	73	69
**Ingestion rate (IR_{ing})[a]**	mg/day	100	100
**Exposure frequency (EF)[a]**	days/year	177	177
**Exposure duration (ED)[a]**	years	65	65
**Average life span (AT)[a]**	days	LT×365	LT×365
**Lifetime (LT)[b]**	years	68.2	78.2
**Surface area (SA)[a]**	cm^2/day	5,700	5,700

**surface factor (SL)[a]**	mg/cm^2	0.07	0.07
**absorption factor (ABS)[a]**	day^{-1}	0.13	0.13
**Inhalation rate (IR_{Inh})[a]**	m^3/day	20	20
**Particle emission factor (PEF)[a]**	m^3/kg	1.36×109	1.36×109

[a] U.S. EPA, 2010. [b] Federal state statistics service.

Table S4. Daily weather conditions in each sample at Vladivostok.

Day	Temperature (℃)	Humidity (%)	Mean wind speed (m/s)
2019/12/21	-5.5	69	4.0
2019/12/22	-11.7	72	6.2
2019/12/23	-12.8	64	5.8
2019/12/24	-12.4	44	3.6
2019/12/25	-9.5	87	4.0
2019/12/26	-14.9	78	8.5
2019/12/27	-16.6	69	7.0
2019/12/28	-12	61	4.7
2019/12/29	-2.9	89	5.4
2020/1/20	-9.7	45	6.1
2020/1/21	-9.2	46	3.1
2020/1/22	-5.4	59	5.1
2020/1/23	-8.3	46	7.8
2020/1/24	-9.9	50	5.1
2020/1/25	-9.2	61	7.9
2020/1/26	-7.9	45	6.8
2020/2/21	2.1	35	2.4
2020/2/22	2	33	4.6
2020/2/23	0.1	56	5.5
2020/2/24	3.4	29	6.0
2020/2/25	0.3	20	4.0
2020/2/26	-1.6	31	4.6
2020/2/27	-0.5	67	4.3
2020/6/19	15.4	89	5.1
2020/6/20	11.7	97	5.8
2020/6/21	14.3	90	4.3
2020/7/22	18.9	-	3.6
2020/7/23	21	94	3.3
2020/7/24	20.1	99	8.0
2020/7/25	17.5	99	4.6
2020/7/26	16.6	-	4.1
2020/7/27	19.6	98	5.6
2020/7/28	20.4	99	7.6

Table S5. Spearman correlation coefficient (two-tailed) of each PAHs and NPAHs with weather condition during sampling period.

Pollutant	Temperature	Wind speed	humidity
**FR**	-0.881	0.203	-0.331
**Pyr**	-0.863	0.180	-0.341
**BaA**	-0.843	0.110	-0.362
**Chr**	-0.845	0.132	-0.367
**BbF**	-0.833	0.103	-0.323
**BkF**	-0.811	0.093	-0.344
**BaP**	-0.802	0.058	-0.370
**BeP**	-0.798	0.084	-0.318
**BgPe**	-0.812	0.069	-0.360
**IDP**	-0.800	0.079	-0.353
**ΣPAHs**	-0.824	0.121	-0.352
**2-NFR**	-0.652	0.001	-0.467
**1-NP**	-0.887	0.166	-0.347
**2-NP**	-0.558	0.015	-0.210
**6-NC**	-0.756	0.112	-0.219
**7-NBaA**	-0.747	0.044	-0.299
**6-NBaP**	-0.808	0.093	-0.347
**ΣNPAHs**	-0.788	0.071	-0.325

The sample size is 35. The correlation coefficient (in bold) between PAHs or NPAHs and wind speed indicates a statistical significance of the correlation with a p-value of less than 0.01. The correlation coefficients between PAHs or NPAHs and wind speed , humidity , indicate no significance of the correlation with a p-value of more than 0.05.

Reference

1. US EPA *U.S. Environmental Protection Agency (US EPA) Benchmark Dose Software. Version 1.10*; **2013**;
2. Collins, J.F.; Brown, J.P.; Alexeeff, G.V.; Salmon, A.G. Potency Equivalency Factors for Some Polycyclic Aromatic Hydrocarbons and Polycyclic Aromatic Hydrocarbon Derivatives. *Regulatory Toxicology and Pharmacology* **1998**, *28*, 45–54, doi:10.1006/rtph.1998.1235.
